# Predicting future dementia from routine clinical MRI and linked healthcare data

**DOI:** 10.1186/s13195-026-02079-4

**Published:** 2026-05-21

**Authors:** Parminder Singh Reel, Salim Al-Wasity, Craig Edwards, Smarti Reel, Esma Mansouri-Benssassi, Szabolcs Suveges, Muthu Rama Krishnan Mookiah, Susan Krueger, Emanuele Trucco, Emily Jefferson, Alexander Doney, J. Douglas Steele

**Affiliations:** 1 Division of Population Health and Genomics, University of Dundee, Dundee, UK; 2 Health Informatics Centre, University of Dundee, Dundee, UK; 3 Division of Neuroscience, University of Dundee, Dundee, UK; 4 College of Engineering, University of Wasit, Kut, Iraq; 5 School of Science and Engineering, Computing, VAMPIRE Project, University of Dundee, Dundee, UK; 6 Health Data Research UK, London, UK

**Keywords:** Dementia risk prediction, Magnetic resonance imaging (MRI), Machine learning, Early detection, Population health

## Abstract

**Background:**

Early identification of individuals at risk of dementia is essential for preventive care and timely enrolment into disease-modifying interventions. However, most existing prediction approaches rely on invasive, costly, or research-only biomarkers that are not scalable within public healthcare systems. Routinely acquired National Health Service (NHS) brain magnetic resonance imaging (MRI) scans, when linked with electronic health records, represent a widely available and privacy-preserving resource for population-level dementia risk stratification. A key challenge for clinical translation is ensuring that machine-learning predictions are reliable, interpretable, and safe to apply, particularly when models are used years before clinical diagnosis.

**Methods:**

We conducted a retrospective case–control study entirely within a secure NHS Trusted Research Environment using routine T1-weighted brain MRI scans linked to electronic health records from Tayside and Fife, Scotland. The study included 518 participants: 259 individuals who subsequently developed dementia and 259 age- and sex-matched controls. Structural brain features were derived from MRI data and analysed using a support-vector-machine classifier with nested cross-validation to minimise overfitting. Prediction confidence was quantified using distance-from-hyperplane (DFH) calibration, enabling stratification of model outputs by certainty. Primary outcomes were classification accuracy and area under the receiver-operating-characteristic curve (AUC). Secondary analyses examined DFH-stratified performance and the relationship between prediction accuracy and time from scan to first recorded dementia diagnosis.

**Results:**

The model predicted future dementia up to five years before first recorded NHS diagnosis with an AUC of 0.71, a performance consistent with real-world clinical imaging rather than research-optimised datasets. Model sensitivity increased for scans acquired closer to diagnosis, indicating stronger predictive signal as disease onset approached. Confidence-based stratification identified a high-confidence subgroup comprising approximately 35% of scans, within which prediction accuracy increased to around 80%. Performance was consistent across heterogeneous routine NHS scanners and imaging protocols, demonstrating robustness and generalisability to real-world clinical data rather than research-optimised acquisitions.

**Conclusion:**

Routinely collected NHS brain MRI data can be used to predict future dementia several years before clinical diagnosis. Incorporating confidence calibration transforms a conventional machine-learning classifier into a safety-aware and clinically interpretable framework by enabling selective use of high-certainty predictions. This approach supports scalable early detection, population-level risk stratification, and targeted recruitment into preventive or disease-modifying clinical trials, with clear potential for integration into public health systems.

**Supplementary Information:**

The online version contains supplementary material available at 10.1186/s13195-026-02079-4.

## Background

Dementia poses one of the most pressing global health challenges, affecting more than 55 million people worldwide and projected to nearly triple by 2050 as populations age [1, 2]. In the UK, dementia care already exceeds £34 billion annually [3], imposing major economic, healthcare, and social burdens. Beyond these costs, the disease carries profound personal and societal consequences [4]. Alzheimer’s disease (AZ) accounts for 60–80% of cases [5], followed by vascular dementia (VD) (~ 20%), and despite decades of research, effective disease-modifying treatments remain limited.

Once clinical symptoms emerge, substantial and irreversible neuronal loss has typically occurred, rendering interventions less effective. Recent advances in disease-modifying therapies for AZ, such as amyloid-targeting monoclonal antibodies, highlight the importance of identifying individuals at pre-symptomatic or prodromal stages [6, 7]. However, early diagnosis remains difficult: current methods rely on specialized cognitive assessments and invasive or costly biomarkers, including PET imaging and cerebrospinal fluid analyses [8], which limit scalability for routine use. Although blood-based biomarkers show promise [9], they have yet to achieve widespread clinical implementation.

Magnetic resonance imaging (MRI) provides a rich, non-invasive source of information about brain structure and health. Machine learning (ML) applied to MRI has shown considerable promise for detecting subtle neurodegenerative patterns that precede dementia symptoms [10–12]. Deep learning and support-vector-machine (SVM) models can distinguish Alzheimer’s disease, mild cognitive impairment, and healthy aging with high accuracy [13]. However, most models rely on research-grade imaging datasets that are carefully curated and homogeneous. These datasets do not represent real-world healthcare populations, which limits the generalizability of such models and their potential for deployment within health systems.

Dementia therefore represents both an urgent clinical need and an opportunity for scalable, data-driven risk prediction. Routinely acquired National Health Service (NHS) brain scans, if effectively leveraged, could enable population-wide dementia risk modelling within privacy-preserving Trusted Research Environments (TREs). Unlike research datasets, these scans capture the heterogeneity of clinical imaging and could support large-scale, real-world implementation.

The present study addresses this opportunity by demonstrating, for the first time, that future dementia can be predicted up to five years before its first recorded NHS diagnosis using routinely collected MRI data. We further introduce a distance-from-hyperplane (DFH) confidence calibration method that quantifies model certainty. This approach transforms a conventional classifier into a clinically interpretable and safety-aware decision-support tool suitable for early intervention triage and precision trial recruitment.

Leveraging longitudinal healthcare data from the NHS provides a transformative approach to dementia research and patient care [14], enabling long-term tracking of disease risk and progression [15]. Integrating such data with routine imaging, for example through national repositories such as the Scottish Medical Imaging (SMI) resource [14], creates a strong foundation for developing predictive models that reflect real-world practice. However, this approach introduces challenges that include diagnostic labelling, data harmonization, quality control, and secure computation. These requirements must be met within Trusted Research Environments, or “Safe Havens,” that safeguard patient privacy [16, 17].

To address these challenges, we conducted a retrospective, case–control study of routinely acquired NHS brain MRI scans linked to longitudinal electronic health records within a secure TRE. Using a nested cross-validated SVM framework, we trained and tested models to distinguish individuals who later developed dementia from matched controls with no dementia diagnosis. Structural brain variability was captured through established neuroanatomical deformation metrics derived from standard clinical T1-weighted images, allowing analysis across heterogeneous scanners typical of NHS practice.

To enhance clinical reliability, DFH-based stratification was applied to quantify model confidence and enable selective prediction of high-certainty cases. This strategy provides an interpretable balance between predictive precision and population coverage. The findings demonstrate the feasibility of developing population-scalable dementia risk prediction tools using routine healthcare imaging data.

## Methods

### Data sources

We combined two previously established research healthcare datasets managed by Health Informatics Centre (HIC) at the University of Dundee which have been described previously: GoDARTS (Genetics of Diabetes Audit and Research in Tayside Scotland) [18] and SHARE (Scottish Health Research register) [19]. All available MRI brain scans collected as part of routine NHS medical practice for Tayside and Fife participants in these studies were obtained from the SMI [14].

### Workflow design

**Fig. 1:**
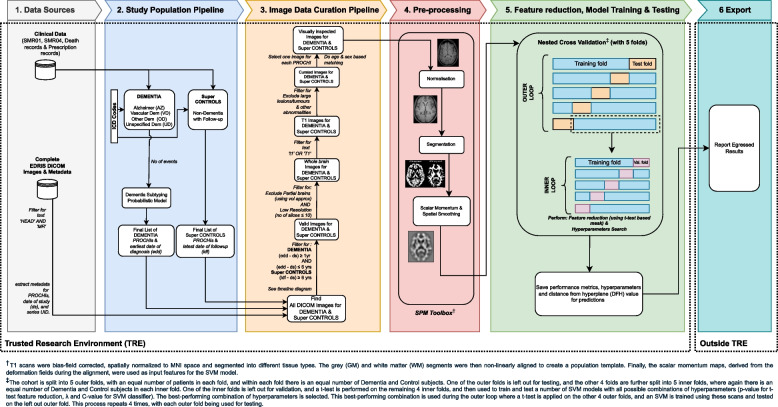
End-to-end six-staged workflow for using routine clinical MRI brain scans and linked prospective electronic medical records (EMR) to predict future dementia using SVM Approach

Figure 1 illustrates an end-to-end six-staged workflow: accessing routinely collected healthcare data and images, constructing study populations, image data curation, pre-processing and support vector machine (SVM)-based ML pipeline and result egress within the TRE environment. Each of these stages is described below.

### Study population pipeline

Using our validated electronic medical record algorithm [20] cases of AZ and VD were identified. In addition, two other groups of cases were identified: “Other dementias” (OD) that include a variety of less common types of dementia, e.g., Lewy body dementia and frontotemporal dementia and “Unspecified dementia” (UD), when the algorithm could not definitively classify the dementia type. Non-dementia controls were identified as patients who did not ever develop dementia. For further details of dementia cases and non-dementia controls definitions see Method S1 and Table S1.

### Image Data Curation Pipeline (IDCP)

This pipeline incorporates a sequential series of filtering steps applied to the image files and DICOM meta-data linked to the study population. Its purpose was to select MRI scans suitable for inclusion in the ML training. Metadata were first filtered for ‘T1’, ‘MR’, and ‘Head’ tags and time window applied: 1–6 years pre-diagnosis for cases, and a minimum of 6 years follow-up after the date of scan or non-dementia death for controls (see Figure S1). A custom annotation tool (see Figure S2) enabled manual review in orthogonal planes to exclude scans with artefacts (e.g., partial brains using volume calculator, see Figure S3) or anatomical lesions). Age and sex were matched across groups. Final scans were converted to NIfTI format. For further details of IDCP see Method S2.

### Preprocessing

All T1-weighted magnetic resonance scans from IDCP were pre-processed using SPM12 [21], running under MATLAB™ R2021a [22]. Scans underwent bias-field correction, spatial normalization to the MNI template, and tissue segmentation into grey matter (GM), white matter (WM), and cerebrospinal fluid (CSF) as shown in Fig. 1. Nonlinear within-subject registration was then performed using the SPM12 Geodesic Shooting Toolbox [23], iteratively refining a population template from segmented GM and WM images. Deformations were computed and applied until a stable, representative template was achieved. Finally, ‘scalar momentum’ [24] was calculated to capture deformation fields, tissue density differences, and morphological variations that provides a rich structural representation to enhance machine learning performance. For further details see Method S3.

### Nested cross-validation

Nested cross-validation was used with the dataset split into five outer folds, with one-fold for testing and the remaining four for ML model training. Within the training set, five inner folds enabled hyperparameter tuning. Feature reduction was performed using a variable-threshold t-test approach within a multivariate pattern analysis framework as discussed below:

#### Feature reduction

To prevent overfitting from high-dimensional data, this study used voxel-wise feature reduction via a two-sample t-test within a nested cross-validation framework. This method selects informative voxels while discarding noisy or redundant voxels, enhancing classification performance and reducing computational cost. The t-test was applied only to training data to avoid data leakage, produced a t-map indicating voxel significance between groups, avoiding double dipping. A binary t-mask was created using an optimal p-value threshold, selected at the same time as optimal SVM hyperparameters. This t-mask was applied to the test set to assess model performance across thresholds, improving generalisability. An additional benefit of using this method was that it explicitly identifies brain regions which were used for predictions, thereby enhancing model transparency. For further details see Method S4.

#### Multi-variate pattern analysis

Radial Basis Function (RBF) SVM as implemented in MATLAB [22] was chosen for its effectiveness with high-dimensional neuroimaging data and superior performance on small datasets compared to deep learning models [13]. The nested cross-validation framework optimized key hyperparameters: box-constraint “C”, and kernel width “λ”, at the same time as p-value threshold for t-test threshold selection, using predefined ranges and a cloud-based SLURM computing (see Method S5). Feature reduction was applied only to training data, generating t-masks for voxel selection, which were then fixed for testing. During training model performance was evaluated on the inner validation folds, and the best hyperparameter set was used for the outer fold as shown in Fig. 1. Final accuracy was averaged across all outer folds.

#### Improving Classification Reliability with SVM DFH (exploratory analysis)

To enhance clinical reliability, we implemented an SVM ‘reject option’ using distance-from-hyperplane (DFH) [25] as a principled measure of prediction confidence, consistent with established safety-aware machine-learning frameworks. This is especially important in medical settings, where misclassification can have serious consequences [26]. DFH provides a measure of prediction confidence, helping clinicians assess individual patient risk of future dementia. Accuracy was calculated by rejecting predictions with DFH below a set threshold, focusing on more confidently classified cases [27, 28]. Performance metrics were evaluated across DFH thresholds from 0 to 1 in 0.05 increments, allowing analysis of trade-offs between confidence, accuracy, and scan coverage.

### Export

All results were securely stored within the TRE and submitted for egress. The TRE administrator conducted disclosure checks [29], redacting sensitive data. Approved result files were then used for reporting in this study.

## Results

### Cohort identification pipeline

**Fig. 2:**
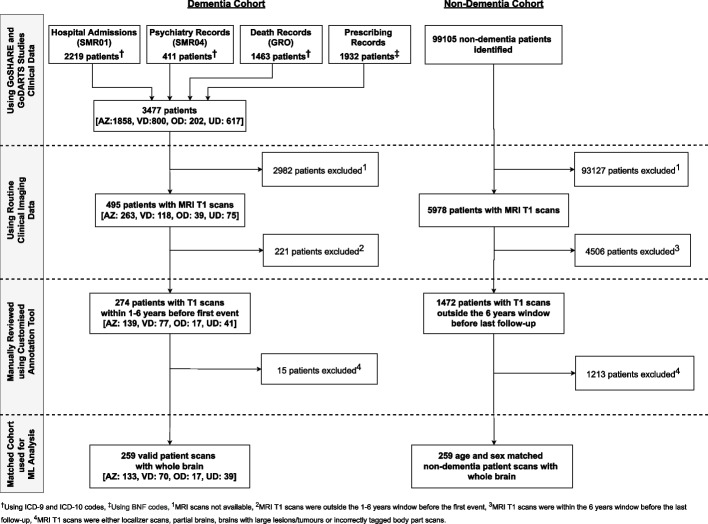
Patient cohort identification and data linkage utilising available EHR and imaging data

Overall, 3,477 patients (AZ [*n* = 1,858], VD [*n* = 800], UD [*n* = 617], and OD [*n* = 202]) who had a diagnosis of dementia in their medical records were identified within the source population (see Fig. 2 and Method S6), and 99,105 patients who never developed such a diagnosis.

### Study population and Image data curation pipeline

In total, 495 dementia cases (AZ [*n* = 263], VD [*n* = 118], UD [*n* = 75], and OD [*n* = 39]) had linked T1 MRI brain scans in routine clinical imaging data. Finally, 259 valid case scans (AZ [*n* = 133], VD [*n* = 70], UD [*n* = 39], and OD [*n* = 17]) were identified after IDCP evaluation. These were age and sex matched with 259 control scans (manually reviewed and validated for any exclusions post-age and sex matching), corresponding to valid case scans. As expected, NHS scans were unlike research scans as they had been acquired using heterogenous scanner parameters (see details in Figure S4). Table 1 provides the population characteristics for this matched study cohort.

**Table 1:** Demographic and clinical characteristics of the study groups

	**Dementia Cases**				**Total Dementia**	**Controls (Non-dementia)**	**Overall**
	Alzheimer's disease (AZ)	Vascular Dementia (VD)	Other Dementia (OD)	Unspecified Dementia (UD)			
Patients (n)	133	70	17	39	259	259	518
***Demographics***							
Age, years (mean ± SD)	72.8 ± 9.0	74.6 ± 7.1	63.4 ± 17.9	76.5 ± 9.3	73.2 ± 9.83	72.6 ± 9.31	72.9 ± 9.6
Sex, female (%)	54.1	37.1	35.3	41.0	46.3	46.3	46.3

### Feature reduction

**Fig. 3:**
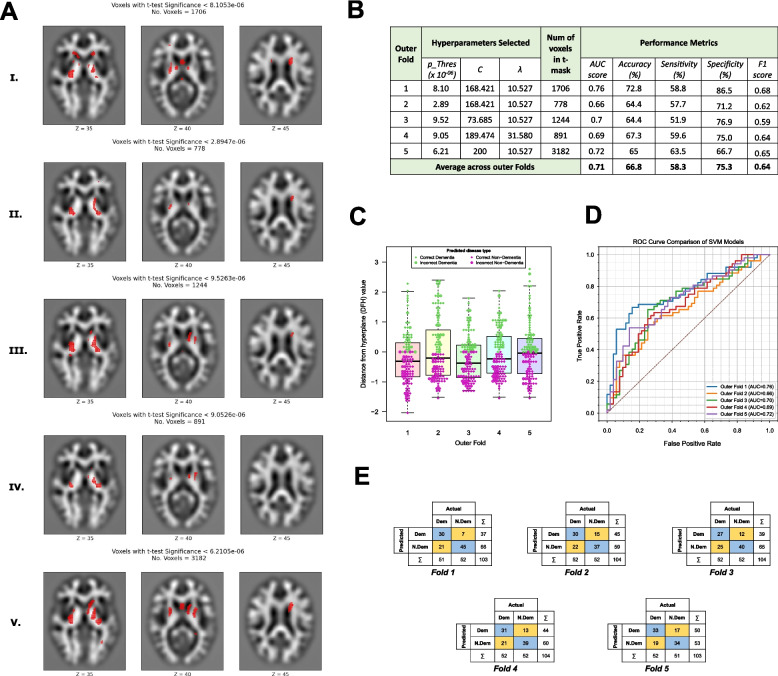
**A** Significant Voxels Overlaid on COLIN Brain Scan for (I) Fold 1, (II) Fold 2, (III) Fold 3, (IV) Fold 4 and (V) Fold 5. The COLIN Brain Scan was pre-processed by segmenting into grey matter and smoothed using scalar momentum. The three scans in each row display different axial slices, inferior to superior across from left to right. **B** Overall summary of performance metrics; **C** Distribution of DFH value for prediction outcomes; **D** ROC curves comparison of SVM models and **E** Confusion matrices across fivefold nested cross-validation

A higher number of most significant voxels were selected as the p-value thresholds (*p_Thres*) gradually increased during the feature reduction in fivefold nested cross validation as illustrated in Figure S5. The final selected t-mask regions used for the outer folds were very similar. Figure 3 (A) shows the final selected t-mask used for each outer fold overlaid on publicly accessible brain scan conforming to standard Montreal Neurological Institute (MNI) anatomical space. The highlighted voxels show regions where multivariate shape differences contribute most strongly to discrimination. The map should therefore be interpreted as the anatomical distribution of distributed predictive shape features, rather than a univariate map of localised atrophy.

### MVPA Performance

#### Analysis 1: All data

The RBF-SVM model performance across five outer folds of the nested cross-validation demonstrated an average AUC score of 0.71 and accuracy of 66.8% in discriminating between scans labelled as future dementia cases and controls as illustrated in Fig. 3 (B). Sensitivity, specificity and F1 score averaged 58.3%, 75.3% and 0.64 respectively, indicating a slightly better ability to correctly identify true positives than true negatives. The number of voxels selected by the t-mask varied widely across folds (ranging from 778 to 3182), reflecting variability in the feature reduction process. Selected hyperparameters also varied, with *p_Thres* ranging from *2.89 to 9.52 (*× *10⁻⁶)*, C values from *73.68* to *200*, and λ values from *10.52* to *31.57*. The full grid search performance across hyperparameters highlight a smooth function as shown in Figure S6. Overall, these results suggest that while the model maintained consistently moderate performance, there was some variability in optimal parameter settings and feature reduction across folds, as expected. Figure 3 (C) shows the distribution of DFH values for prediction outcomes across each fold.

Figure 3 (D) shows the ROC curves comparison of SVM models for the 5 outer folds. The confusion matrices across five folds show reasonable consistency in the model’s classification of two classes, with varying strengths across folds as shown in Fig. 3 (E). Fold 1 delivers the best balance between sensitivity and specificity, while Folds 2 and 3 show reduced specificity and sensitivity due to increased false predictions. Fold 4 improves this balance slightly, and Fold 5 maintains reasonable accuracy but with a higher false positive rate. Overall, the model performs reliably but exhibits some variability across folds, indicating potential benefits from further tuning or regularization to enhance generalisation.

#### Analysis 2: Filtered data

**Fig. 4:**
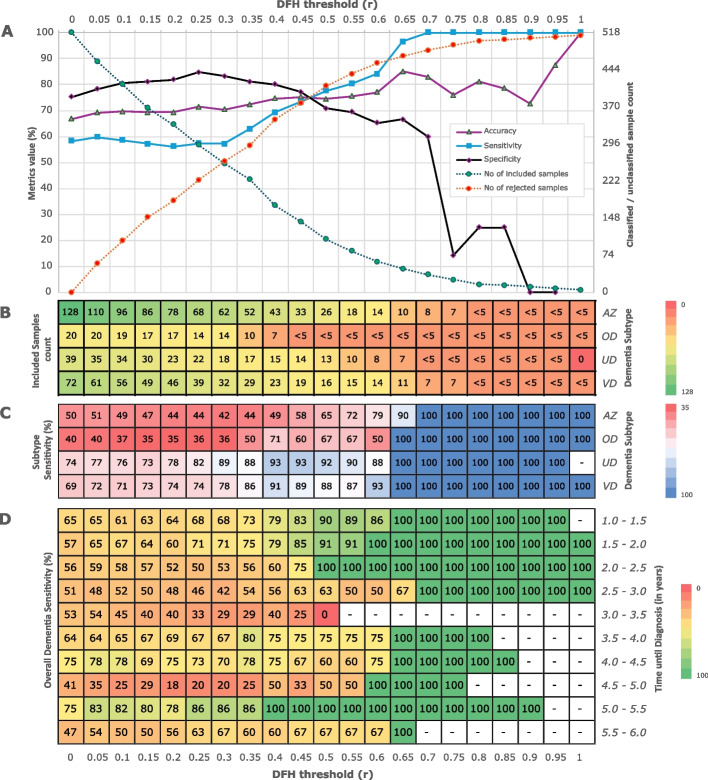
**A** Classification metrics (accuracy, sensitivity, and specificity) with varying DFH threshold, **B** Classified sample count across dementia subtype, **C** Sensitivity (%) across dementia subtypes and **D** overall dementia sensitivity w.r.t time until diagnosis (in years)

Figure 4 (A) shows varying the DFH threshold (ranging from 0 to 1 in 0.05 increments) impacts classification performance, showing a trade-off between sample coverage and classification metrics. As the threshold increases from 0 to 1, the number of classified samples dropped steadily (from 518 to 5), while accuracy and sensitivity generally improved. Early thresholds (0–0.3) prioritise broader classification, with more balanced sensitivity and specificity. From thresholds 0.4 to 0.65, the model achieves a notable balanced sensitivity, specificity while maintaining moderate classification coverage. Beyond 0.7, sensitivity reached 100% as the model became highly conservative, only predicting when very “confident” but at the cost of reduced specificity and sample coverage. At threshold = 1, all classified predictions are correct (100% accuracy), but the sample size was minimal, making performance estimates less reliable. This trade-off illustrates the importance of selecting a threshold that balances confidence with meaningful data coverage for the intended application.

##### Prediction of dementia subtypes

The model demonstrated promising results in identifying patients who later developed specific dementia subtypes. Figure 4 (B—C) presents the dementia subtype (AZ, OD, UD, and VD) count and their sensitivity values w.r.t increasing DFH thresholds. As the threshold increases from 0 to 1, the total number of included samples decreases across all subgroups. Correspondingly, sensitivity increases in all categories, indicating improved classification performance with higher-quality inputs. At threshold 0, sensitivities are relatively low to moderate (AZ: 50%, OD: 40%, UD: 74.4%, VD: 69.4%). However, by threshold 0.65, all sensitivity values reach or exceed 90%, with most achieving 100% by 0.7. This trend illustrates a clear trade-off between sample size and model sensitivity: excluding lower-confidence cases enhances performance metrics but reduces the sample size significantly. For thresholds above 0.75, sensitivities remain at 100% for all applicable categories, although the number of retained cases becomes small.

### Prediction of time for individual patient’s to develop dementia

Figure 4 (D) illustrates dementia prediction sensitivity as a function of the time until patients received their diagnosis. Overall, the results demonstrate that applying higher DFH thresholds improves sensitivity across nearly all time intervals, particularly in the earlier stages of disease progression. In the short-term intervals (1.0–2.5 years), sensitivity is already relatively high at baseline (e.g., 1.0–1.5 years: 65%, 1.5–2.0 years: 57%, 2.0–2.5 years: 56%) and increases steadily with higher thresholds, reaching 100% sensitivity by thresholds 0.65–0.75 in most cases. This indicates the model’s strong ability to detect imminent diagnoses under stricter threshold settings. Mid-range intervals (2.5–4.5 years) exhibit greater variability in sensitivity. For example, in the 2.5–3.0 year group, sensitivity fluctuates between 42–63% across thresholds, only reaching 100% at threshold 0.65. The 3.0–3.5 year group shows a marked decline in sensitivity beyond threshold 0.3, with values dropping to 0% at threshold 0.5 and absent thereafter, reflecting reduced model reliability in this interval. In contrast, the 4.0–4.5 year interval maintains relatively high sensitivity (60–78%) at lower thresholds and reaches full sensitivity by 0.6. For longer-term intervals (4.5–6.0 years), sensitivity varies widely. In the 4.5–5.0 year range, values are low at lower thresholds (as low as 18%) but improve to 100% by threshold 0.6. Similarly, the 5.0–5.5 and 5.5–6.0 year groups achieve full sensitivity at thresholds 0.45 and 0.65, respectively, although data becomes sparse or missing beyond those points. Overall, increasing the DFH threshold consistently enhances sensitivity, particularly in early and mid-term diagnosis windows, but often at the expense of sample coverage in longer-term cases.

## Discussion

This study demonstrates that future dementia can be predicted up to five years before the first recorded NHS diagnosis using routinely acquired clinical MRI brain scans linked to electronic health records within a privacy preserving Trusted Research Environment. By analysing data collected during standard clinical care rather than research protocols, we show the feasibility of developing population scalable predictive models capable of operating within real world imaging heterogeneity. This establishes a critical proof of principle that routinely available NHS imaging data can support large scale, prospective dementia risk stratification without the need for new data collection or bespoke imaging sequences. A second major contribution is the introduction of a distance from hyperplane confidence calibration approach, which quantifies the certainty of each individual patient machine learning prediction. This strategy allows the model to prioritise high confidence classifications, achieving approximately 80 percent accuracy for one third of scans while maintaining a consistent baseline performance with AUC equal to 0.71 across the entire dataset. Rather than a post hoc adjustment, distance from hyperplane stratification transforms the classifier into a selective prediction system analogous to clinical reasoning, where uncertain cases can be deferred or referred for further evaluation. This capability enhances interpretability, patient safety, and clinical trust, properties increasingly recognised as essential for the responsible deployment of artificial intelligence systems in healthcare. Taken together, these findings establish three interlocking advances. First, demonstration that routine NHS radiology can underpin national scale dementia risk modelling. Second, evidence that future dementia can be predicted years in advance of NHS diagnosis. Third, introduction of confidence calibrated prediction enabling safe translation from research models to clinical decision support. These innovations collectively provide a foundation for scalable early detection and trial recruitment frameworks across the NHS and comparable health systems.

The use of routinely collected scans introduced challenges in cohort identification and image curation. These real-world clinical scans required exclusions for artefacts. All data access was within a TRE, where scans were pseudonymised and securely accessed. This setup required modifications to the ML development cycle [30], including stringent disclosure controls [31], ensuring data security and ethical use. This study focused on predicting future dementia diagnosis when brain changes are more likely to be subtle. In contrast, many high-accuracy studies [32] predict dementia at a cross-sectional diagnostic stage, when structural abnormalities are pronounced. Furthermore, we used an age-matched dataset to ensure we were not just predicting older age. The field of dementia diagnosis using brain imaging has used various ML methods [33], especially SVM, with studies reporting substantially higher accuracies [34–37] typically rely on research-grade imaging, tightly controlled cohorts, or near-diagnostic timepoints. When restricted to routinely acquired clinical scans such as NHS and multi-year pre-diagnostic prediction is the goal, reported performance converges with the range observed here.

Our study further confirms that predictive sensitivity improves as the time to clinical diagnosis decreases, with peak sensitivity observed between 1.5 and 2 years prior. This aligns with earlier findings [38–40] that show Alzheimer’s disease can be detected 3–4 years before clinical diagnosis. Our results support the idea of a "silent phase" in dementia progression, during which early structural brain changes (e.g., atrophy and white matter loss) occur before cognitive symptoms manifest [41]. Our accuracy levels are commensurate with a range of other studies reporting moderate to high dementia classification performance using a range of different risk models (not involving brain imaging) for predicting future dementia [32].

The date of dementia diagnosis used in this study was derived from a validated algorithm [20] which integrates information from multiple NHS data sources including primary care, secondary care, prescribing, and mortality records, to estimate when an NHS clinician first recognised dementia. Because no single gold-standard repository of dementia diagnoses exists within UK health data, this composite algorithm provides the best available real-world approximation of clinical diagnosis. Accordingly, predicting years in advance of this algorithm-derived date represents a genuine gain in lead time before dementia would be identified in real world NHS practice, creating a clinically meaningful window for early assessment or intervention.

There are currently no treatments that reverse Alzheimer’s or vascular dementia. The therapeutic goal is to slow or stop progression, making early prediction critical. Accurate identification of high-risk individuals enables timely intervention. For vascular dementia, existing strategies such as smoking cessation and blood pressure or lipid control can be implemented [42]. For Alzheimer’s disease, new disease-modifying drugs are emerging [43], but their success depends on early, accurate patient identification. Given the high prevalence of dementia, prediction methods must use existing routinely acquired clinical scans, as collecting new data at scale is unfeasible at a population level. Many failed Alzheimer’s drug trials have cited the inability to identify patients early enough as a key reason for failure [44]. Our method addresses this inability and could help recruit large, well-characterised cohorts, for future trials of disease modifying drugs.

A key design choice in this feasibility study was to age- and sex-match dementia cases and controls, deliberately excluding age as a predictor despite its strong association with dementia risk. This approach isolated the independent predictive contribution of the brain scan itself, establishing proof of concept that routinely acquired MRI contains sufficient latent information to forecast future dementia. The intended next stage is to integrate this scan-derived probability and its distance-from-hyperplane confidence with readily available clinical variables, such as age, family history, and, where known, APOE genotype, to create a composite risk calculator analogous to those used in cardiology or oncology. In clinical use, a doctor could obtain a standard NHS brain scan, receive an automated binary prediction with confidence calibration, and then combine this with patient-specific information to derive an overall personalised dementia risk estimate for informed preventive care.

In summary, this study establishes that routinely acquired NHS brain MRI scans contain sufficient prognostic information to predict dementia years before it is clinically recognised. By using a validated, multi-source NHS algorithm to define diagnosis in the absence of a national gold standard, the model predicts meaningfully earlier than an NHS clinician would typically record dementia. The combination of routine imaging, real-world diagnostic timing, and confidence-calibrated prediction provides the foundation for a future composite risk calculator integrating scan-derived probability with readily available clinical variables. Such a system could transform dementia care from reactive diagnosis to proactive, population-scale prevention.

### Clinical and research implications

The results imply that predictive modelling of dementia risk can move beyond research datasets toward real-world, population-scale implementation. The ability to generate accurate individual patient predictions from routine NHS MRI scans further opens the opportunity of integrating machine-learning tools into existing radiology workflows and health-record systems, identifying high-risk individuals long before symptoms emerge. In practice, confidence-calibrated outputs such as DFH enable selective clinical deployment: high-certainty predictions can be actioned, while low-certainty cases are explicitly deferred, mirroring established medical risk-stratification paradigms.

From a health-system perspective, this approach provides a low-cost, scalable route to precision prevention, using data that are already being collected throughout the NHS. Future research should validate the method prospectively in larger, demographically diverse cohorts, assess temporal generalisability across sites, and integrate complementary risk factors such as genetics and blood biomarkers. If confirmed, the combination of routine NHS radiology, population linkage, and confidence-calibrated AI could enable a paradigm shift from symptomatic diagnosis to proactive, pre-symptomatic dementia prevention.

### Limitations

Despite these promising findings, several limitations should be noted. As a feasibility study using a regional Scottish population, the sample size was limited, potentially affecting model generalizability. Routinely collected scans showed heterogeneity in image parameters and equipment, but this was necessary for scaling to a population level. The control group consisted of patients with non-dementia health conditions rather than a healthy population, potentially affecting specificity, but likely more realistic for clinical practice. We also grouped four dementia subtypes together for classification, which improved sample size but may have reduced subtype-specific insights. Confidence intervals were not estimated due to the nested cross validation framework; however, variability across outer folds is reported to provide an indication of model stability and uncertainty. We did not account for time-to-diagnosis in model training, possibly masking temporal prediction dynamics. Future studies, such as the upcoming SCAN-DAN project [45], will apply these cohort and data curation pipelines to a larger, nationwide Scottish dataset. This will help refine the model and validate its performance across more diverse populations.

## Conclusion

In conclusion, this study presents a novel ML pipeline that utilizes routinely collected clinical scan data, showing considerable promise for predicting future dementia diagnosis, subtype prediction, and timeline forecasting. These findings lay the groundwork for future research, particularly the SCAN-DAN study, which will leverage the same cohort identification and data curation pipelines on a national scale. This expanded scope is expected to refine the model further, moving closer to a clinically translatable tool for predicting later dementia diagnosis with subtype differentiation.

## Supplementary Information

Supplementary Material 1. Method S1: Cases and controls identification using EMR datasets. Method S2: Identifying valid brain scans. Method S3: Preprocessing valid brain scans. Method S4: Feature reduction. Method S5: Hyper-parameter details. Method S6: Patient cohort identified using EMR datasets. Table S1: Selection of ICD-9 and ICD-10 codes used to select different dementia types. Figure S1: Example of time window showing the timeline of events used for selecting valid scans. Figure S2: Snapshot showing GUI of bespoke annotation tool developed in RShiny and used to manually inspect and annotate brain scans. Figure S3: Flowchart showing the volume calculation to filter partial brain scans. Figure S4: Summary of sequence parameters for T1w MRI images. Where TR: repetition time, TE: echo time. Figure S5: Number of voxels selected during feature reduction in 5-fold nested cross-validation. Figure S6: Heatmap showing grid search performance across hyperparameters.

## Data Availability

The data related to the results presented in this article can be accessed within the HIC TRE subject to ethical and governance approvals. The details of dementia cohort identification can be accessed online on the HDR UK Phenotype library (https://phenotypes.healthdatagateway.org/phenotypes/PH1717/version/3973/detail/). The dementia cohort identification and SVM codebase used in this study is available online at GitHub (https://github.com/HicResearch/PICTURES-DementiaClassifier).

## Abbreviations

AUC: Area under the receiver-operating-characteristic curve
AZ: Alzheimer’s disease
CSF: Cerebrospinal fluid
DFH: Distance-from-hyperplane
GM: Grey matter
GoDARTS: Genetics of Diabetes Audit and Research in Tayside Scotland
HIC: Health Informatics Centre
IDCP: Image Data Curation Pipeline
ML: Machine learning
MNI: Montreal Neurological Institute
MRI: Magnetic resonance imaging
NHS: National Health Service
OD: Other dementias
p_Thres: P-value thresholds
RBF: Radial Basis Function
SHARE: Scottish Health Research register
SMI: Scottish Medical Imaging
SVM: Support vector machine
TREs: Trusted Research Environments
UD: Unspecified dementia
VD: Vascular dementia
WM: White matter

## References

[1] Nichols E, Steinmetz JD, Vollset SE, et al. Estimation of the global prevalence of dementia in 2019 and forecasted prevalence in 2050: an analysis for the Global Burden of Disease Study 2019. Lancet Public Health. 2022;7:e105–25. 34998485 10.1016/S2468-2667(21)00249-8 PMC8810394

[2] Facts for the media about dementia | Alzheimer’s Society, https://www.alzheimers.org.uk/about-us/news-and-media/facts-media (2024, accessed 18 August 2025).

[3] Livingston G, Huntley J, Sommerlad A, et al. Dementia prevention, intervention, and care: 2020 report of the Lancet Commission. Lancet. 2020;396:413–46. 32738937 10.1016/S0140-6736(20)30367-6 PMC7392084

[4] Wittenberg R, Knapp M, Hu B, et al. The costs of dementia in England. Int J Geriatr Psychiatry. 2019;34:1095–103. 30950106 10.1002/gps.5113 PMC6618309

[5] Alzheimer’s disease facts and figures. Alzheimer’s & Dementia. 2024;20:3708–821. 10.1002/alz.13809 PMC11095490 38689398

[6] Dubois B, Feldman HH, Jacova C, et al. Advancing research diagnostic criteria for Alzheimer’s disease: the IWG-2 criteria. Lancet Neurol. 2014;13:614–29. 24849862 10.1016/S1474-4422(14)70090-0

[7] Rosenberg A, Solomon A, Soininen H, et al. Research diagnostic criteria for Alzheimer’s disease: findings from the LipiDiDiet randomized controlled trial. Alzheimers Res Ther. 2021;13:64. 33766132 10.1186/s13195-021-00799-3 PMC7995792

[8] Hampel H, O’Bryant SE, Molinuevo JL, et al. Blood-based biomarkers for Alzheimer disease: mapping the road to the clinic. Nat Rev Neurol. 2018;14:639–52. 30297701 10.1038/s41582-018-0079-7 PMC6211654

[9] Grande G, Valletta M, Rizzuto D, et al. Blood-based biomarkers of Alzheimer’s disease and incident dementia in the community. Nat Med. 2025. 10.1038/s41591-025-03605-x. 40140622 10.1038/s41591-025-03605-x PMC12176656

[10] Javeed A, Dallora AL, Berglund JS, et al. Machine learning for dementia prediction: a systematic review and future research directions. J Med Syst. 2023;47:17. 36720727 10.1007/s10916-023-01906-7 PMC9889464

[11] Arbabshirani MR, Plis S, Sui J, et al. Single subject prediction of brain disorders in neuroimaging: promises and pitfalls. Neuroimage. 2017;145:137–65. 27012503 10.1016/j.neuroimage.2016.02.079 PMC5031516

[12] Ebrahimi A, Luo S, Initiative ADN. Convolutional neural networks for Alzheimer’s disease detection on MRI images. J Med Imaging. 2021;8:024503. 10.1117/1.JMI.8.2.024503 PMC8083897 33937437

[13] Wen J, Thibeau-Sutre E, Diaz-Melo M, et al. Convolutional neural networks for classification of Alzheimer’s disease: overview and reproducible evaluation. Med Image Anal. 2020;63:101694. 32417716 10.1016/j.media.2020.101694

[14] Baxter R, Nind T, Sutherland J, et al. The Scottish medical imaging archive: 57.3 million radiology studies linked to their medical records. Radiol Artif Intell. 2024;6:e220266. 38166330 10.1148/ryai.220266 PMC10831519

[15] Li Q, Yang X, Xu J, et al. Early prediction of Alzheimer’s disease and related dementias using real-world electronic health records. Alzheimers Dement. 2023;19:3506–18. 36815661 10.1002/alz.12967 PMC10976442

[16] Bucholc M, James C, Khleifat AA, et al. Artificial intelligence for dementia research methods optimization. Alzheimers Dement. 2023;19:5934–51. 37639369 10.1002/alz.13441

[17] Lea NC, Nicholls J, Dobbs C, et al. Data safe havens and trust: toward a common understanding of trusted research platforms for governing secure and ethical health research. JMIR Med Inform. 2016;4:e22. 27329087 10.2196/medinform.5571 PMC4933798

[18] Hébert HL, Shepherd B, Milburn K, et al. Cohort profile: genetics of diabetes audit and research in Tayside Scotland (GoDARTS). Int J Epidemiol. 2018;47:380–381j. 29025058 10.1093/ije/dyx140 PMC5913637

[19] McKinstry B, Sullivan FM, Vasishta S, et al. Cohort profile: the Scottish research register SHARE. A register of people interested in research participation linked to NHS data sets. BMJ Open. 2017;7:e013351. 28148535 10.1136/bmjopen-2016-013351 PMC5293989

[20] Doney ASF, Bonney W, Jefferson E, et al. Investigating the relationship between Type 2 diabetes and dementia using electronic medical records in the GoDARTS bioresource. Diabetes Care. 2019;42:1973–80. 31391202 10.2337/dc19-0380

[21] SPM - Statistical Parametric Mapping, https://www.fil.ion.ucl.ac.uk/spm/ (accessed 12 October 2024).

[22] MATLAB, https://www.mathworks.com/products/matlab.html (accessed 12 October 2024).

[23] Ashburner J, Friston KJ. Diffeomorphic registration using geodesic shooting and Gauss-Newton optimisation. Neuroimage. 2011;55:954–67. 21216294 10.1016/j.neuroimage.2010.12.049 PMC3221052

[24] Singh N, Fletcher PT, Preston JS, et al. Multivariate Statistical Analysis of Deformation Momenta Relating Anatomical Shape to Neuropsychological Measures. In: Jiang T, Navab N, Pluim JPW, et al., editors. Medical Image Computing and Computer-Assisted Intervention – MICCAI 2010. Berlin, Heidelberg: Springer; 2010. p. 529–37. 10.1007/978-3-642-15711-0_66 20879441

[25] Hendrickx K, Perini L, Van der Plas D, et al. Machine learning with a reject option: a survey. Mach Learn. 2024;113:3073–110.

[26] Liu J, Gallego B, Barbieri S. Incorporating uncertainty in learning to defer algorithms for safe computer-aided diagnosis. Sci Rep. 2022;12:1762. 35110629 10.1038/s41598-022-05725-7 PMC8810991

[27] Fumera G, Roli F. Support Vector Machines with Embedded Reject Option. In: Lee S-W, Verri A, editors. Pattern Recognition with Support Vector Machines. Berlin, Heidelberg: Springer; 2002. p. 68–82.

[28] Drapal P, Silva-Filho T, Prudêncio RBC. Meta-Learning and Novelty Detection for Machine Learning with Reject Option. In: 2024 International Joint Conference on Neural Networks (IJCNN), pp. 1–8.

[29] HIC Team. Data Security - HIC Policies and Standard Operating Procedures - Confluence, https://hicservices.atlassian.net/wiki/spaces/HICSOP/pages/702218242/Data+Security (accessed 26 May 2025).

[30] Reel PS, Aaron J, Krueger S, et al. Designing Machine Learning Experiments using SLURM within a Cloud Trusted Research Environment. Epub ahead of print 6 September 2023. 10.5281/zenodo.13921331.

[31] Jefferson E, Liley J, Malone M, et al. GRAIMATTER Green Paper: Recommendations for disclosure control of trained Machine Learning (ML) models from Trusted Research Environments (TREs). Zenodo. Epub ahead of print 21 September 2022. 10.5281/zenodo.7089491.

[32] Hou X-H, Feng L, Zhang C, et al. Models for predicting risk of dementia: a systematic review. J Neurol Neurosurg Psychiatry. 2019;90:373–9. 29954871 10.1136/jnnp-2018-318212

[33] Twait EL, Andaur Navarro CL, Gudnason V, et al. Dementia prediction in the general population using clinically accessible variables: a proof-of-concept study using machine learning. The AGES-Reykjavik study. BMC Med Inform Decis Mak. 2023;23:168. 37641038 10.1186/s12911-023-02244-x PMC10463542

[34] Kloppel S, Stonnington CM, Chu C, et al. Automatic classification of MR scans in Alzheimer’s disease. Brain. 2008;131:681–9. 18202106 10.1093/brain/awm319 PMC2579744

[35] S. K. Aruna, S. Chitra. Machine Learning Approach For Identifying Dementia From Mri Images. Epub ahead of print 3 April 2016. 10.5281/ZENODO.1124465.

[36] Zheng Y, Guo H, Zhang L, et al. Machine learning-based framework for differential diagnosis between vascular dementia and Alzheimer’s disease using structural MRI features. Front Neurol. 2019;10:1097. 31708854 10.3389/fneur.2019.01097 PMC6823227

[37] Leong LK, Abdullah AA. Prediction of Alzheimer’s disease (AD) using machine learning techniques with Boruta algorithm as feature selection method. J Phys Conf Ser. 2019;1372:012065.

[38] Adaszewski S, Dukart J, Kherif F, et al. How early can we predict Alzheimer’s disease using computational anatomy? Neurobiol Aging. 2013;34:2815–26. 23890839 10.1016/j.neurobiolaging.2013.06.015

[39] Moscoso A, Silva-Rodríguez J, Aldrey JM, et al. Prediction of Alzheimer’s disease dementia with MRI beyond the short-term: implications for the design of predictive models. Neuroimage Clin. 2019;23:101837. 31078938 10.1016/j.nicl.2019.101837 PMC6515129

[40] Franzmeier N, Koutsouleris N, Benzinger T, et al. Predicting sporadic Alzheimer’s disease progression via inherited Alzheimer’s disease-informed machine-learning. Alzheimers Dement. 2020;16:501–11. 32043733 10.1002/alz.12032 PMC7222030

[41] Gabitto MI, Travaglini KJ, Rachleff VM, et al. Integrated multimodal cell atlas of Alzheimer’s disease. Nat Neurosci. 2024;27:2366–83. 39402379 10.1038/s41593-024-01774-5 PMC11614693

[42] Kleindorfer DO, Towfighi A, Chaturvedi S, et al. 2021 guideline for the prevention of stroke in patients with stroke and transient ischemic attack: a guideline from the American Heart Association/American Stroke Association. Stroke. 2021;52:e364–467. 34024117 10.1161/STR.0000000000000375

[43] Researching new drugs for Alzheimer’s disease | Alzheimer’s Society, https://www.alzheimers.org.uk/about-dementia/treatments/researching-new-drugs-alzheimers-disease (accessed 15 May 2025).

[44] Kim CK, Lee YR, Ong L, et al. Alzheimer’s disease: key insights from two decades of clinical trial failures. J Alzheimers Dis. 2022;87:83. 35342092 10.3233/JAD-215699 PMC9198803

[45] AI software tool aims to spot early signs of dementia from brain scans. Edinburgh Innovations, https://edinburgh-innovations.ed.ac.uk/news/ai-software-tool-aims-to-spot-early-signs-of-dementia-from-brain-scans-during-routine-appointments (accessed 11 November 2024).

